# Effectiveness of school-based brief cognitive behavioral therapy with mindfulness in improving the mental health of adolescents in a Japanese school setting: A preliminary study

**DOI:** 10.3389/fpsyg.2022.895086

**Published:** 2022-08-03

**Authors:** Kiun Kato, Yuki Matsumoto, Yoshiyuki Hirano

**Affiliations:** ^1^Research Center for Child Mental Development, Chiba University, Chiba, Japan; ^2^United Graduate School of Child Development, Osaka University, Kanazawa University, Hamamatsu University School of Medicine, Chiba University and University of Fukui, Suita, Japan; ^3^Health Support Center, Waseda University, Tokyo, Japan; ^4^School of Human Life Sciences, Tokushima Bunri University, Tokushima, Japan

**Keywords:** adolescents, brief cognitive behavioral therapy, emotional regulation, mindfulness, school-based program, depression, anxiety, mental health prevention program

## Abstract

**Background:**

Emotional regulation is important for adolescents’ adaptive development. Preventive interventions for anxiety and depression are necessary for reducing the development of disorders later in life, and emotional regulation is a potentially relevant factor.

**Objective:**

We investigated the effects of a mindfulness-based psychological education and prevention program [the Mindfulness and Awareness Program (MAP)] on the mental health of junior high school students in Japan.

**Methods:**

Our MAP primarily focused on mindfulness meditation to improve emotional regulation, thereby reducing depression and anxiety. The MAP comprised eight sessions (20 min each) administered by a school counselor in a school setting. All participants (*N* = 349) were 12–13-year-old adolescents from nine classes in two Japanese schools. The program was provided to the intervention group, wherein students were educated on emotional expression, emotional cognition, and emotional regulation. The control group received regular school counseling services.

**Results:**

Compared with the control group, the intervention group showed significant improvement in emotional regulation and a decrease in depression and generalized anxiety. The effect was greater at the follow-up assessment than at the immediate post-intervention assessment, and greater in female students.

**Conclusion:**

Our mental health prevention program exhibited efficacy in reducing depression and anxiety and enhancing emotional regulation in early adolescence. Further, it appeared to be more effective for female adolescents.

## Introduction

Psychosocial stress is an important risk factor for the internalization (e.g., anxiety and depression) and externalization (e.g., behavioral aspects) of problems in childhood and adolescence (McMahon et al., 2003; Grant et al., 2006). A meta-analysis found that the worldwide prevalence of anxiety disorders was 6.5%, and that of depressive and disruptive disorders was 2.6 and 5.7%, respectively, in children and adolescents (Polanczyk et al., 2015). Moreover, the prevalence of depressive disorders has increased among younger generations (Abela and Hankin, 2008). Depression co-occurs with disorders such as anxiety (Essau et al., 2010).

Japanese adolescents are displaying increased mental health problems. In Japan, 25.9% of junior high school students exhibit high depressive tendencies (Kawakatsu et al., 2014). Using semi-structured interviews, Sato et al. (2008) found that the prevalence of depression in Japanese junior high school students was 4.9% (boys: 2.2%; girls: 8.0%); the reported lifetime prevalence rate was 8.8% (boys: 6.2%; girls: 12.0%). Anxiety among Japanese children and adolescents was estimated as being similar to those in Western societies (Ishikawa, 2013). Additionally, Japanese junior high school students experience considerable interpersonal relationship difficulties and overwhelming feelings of powerlessness (Houri et al., 2012).

Japanese teachers lack the time to promote preventive psychoeducation. According to the Teaching and Learning International Survey by the Organisation for Economic Co-operation and Development, Japanese teachers worked the longest hours per week among the participating countries (56.0 h in Japan, compared with 38.3 h on average in the participating countries; Ministry of Education, Culture, Sports, Science and Technology, 2019). There is a strong need for support staff to provide quality education (Ministry of Education, Culture, Sports, Science and Technology, 2019). School counselors have been appointed in Japanese schools since 1995, and the number of schools with school counselors has been increasing every year due to their activities (Ministry of Education, Culture, Sports, Science and Technology, 2020). The role of school counselors also includes the provision of psychoeducation to students (Ministry of Education, Culture, Sports, Science and Technology, 2020). In Japan, school counselors are expected to lighten the burden of teachers by providing preventive psychoeducation.

Schools are a major venue for delivering mental health services to children (Rones and Hoagwood, 2000), and may provide the ideal context for implementing evidence-based interventions (Eiraldi et al., 2015). Recognizing the prevalence of mental health problems (which include school-related mental health problems) as a serious social threat that thwarts the healthy development and well-being of children has led to Japanese educators’ heightened interest in mental health prevention programs (Matsumoto et al., 2020). However, while the necessity of universal preventive programs in Japanese school settings has been recognized, it has not been systematically implemented due to the lack of psychoeducational programs in the school curriculum (Yamazaki, 2013). Therefore, the program’s duration and efficacy need to be considered in order to disseminate universal preventive programs in Japan (Miyake et al., 2008).

Mindfulness-based interventions (MBIs) are widely used for various clinical and non-clinical purposes and are generally effective in reducing physical and psychological problems, such as anxiety and depression, and improving health and well-being (Hofmann et al., 2010; Keng et al., 2011). Given that students spend the majority of their time in school and many students can be reached directly in the classroom (Weare and Nind, 2011; Zenner et al., 2014), MBIs and prevention programs to support student mental health in the classroom have increased considerably in recent years (Carsley et al., 2018). Although school-based MBIs vary in content and outcomes, there is preliminary evidence supporting their benefits for students’ well-being (Weare, 2013). Reported benefits include increases in indicators of positive emotions such as happiness and optimism after the intervention (Sampaio de Carvalho et al., 2017) and decreases in indicators of negative emotions such as fear and worry (Sibinga et al., 2016). Thus, there is growing interest in the use of MBIs for school students (Felver et al., 2016) as they are effective for managing various psychosocial problems (Khoury et al., 2013).

Few studies have focused on MBIs among junior high school students in Japan. Prior research includes eight reports on mindfulness programs in Japanese schools (Tsuchiya and Koseki, 2017), but none on mindfulness programs for junior high school students. Ashiya et al. (2017) implemented the “.b” program (a mindfulness program developed in the United Kingdom) for sixth-grade students in Japanese elementary schools. They reported three issues: costly and time-consuming training for instructors, the need to devise a program that is adapted to the actual educational situation in Japan, and the need for accumulating research evidence on the effectiveness of MBIs in Japan. Adapting mental health prevention programs to the Japanese educational environment is especially important in terms of their implementation, as the cultural context is different from that of Western countries, where many mental health prevention programs have been developed and studied (Matsumoto et al., 2020). Stallard et al. (2012), in a meta-analysis, revealed that the effectiveness of school-based programs was not consistent across different settings. This can be attributed to the various components and delivery protocols of programs delivered in specific social-cultural contexts (Matsumoto et al., 2020). Therefore, developing MBI programs adapted to the Japanese educational environment and accumulating research evidence on the effectiveness of MBIs in Japan can contribute to the advancement of psychological prevention programs in Japanese schools.

Psychoeducational preventive programs that are acceptable and feasible in the Japanese school environment are required. Brief cognitive behavioral therapy (BCBT) was introduced to provide children with anxiety treatment. This form of cognitive behavioral therapy (CBT) is more practicable in community settings due to its reduced treatment duration. It makes dissemination and implementation more feasible through reduced costs and increased access to services (Beidas et al., 2013). BCBT comprises (a) psychoeducation and (b) skills training (e.g., affect recognition, cognitive restructuring, relaxation, and problem solving; Crawley et al., 2013). One study reported the implementation of a BCBT program that provided insight on emotional, behavioral, and interpersonal problems, and program acceptability in Japanese schools (Matsumoto and Ishimoto, 2015).

In this study, we aimed to develop and assess a program structured around mindfulness for improving emotional regulation skills, adopting the BCBT framework to adapt our mental health preventive program to the Japanese educational environment for adolescents.

The developed program was named the Mindfulness Awareness Program (MAP). Mindfulness meditation facilitates attentional self-regulation and emotional regulation (Kabat-Zinn, 2009). The association between emotional regulation and symptoms of psychopathology has been reported in children and adolescents (Compas et al., 2017). Over the past 30 years, mindfulness techniques, such as body scan and meditation, have reportedly helped to prevent and mitigate emotional distress (Raes et al., 2014).

The MAP can be implemented by a school counselor, reducing the burden on teachers while obtaining their cooperation. As teachers are in a position to ensure consistency and connection with students over time, a number of stress management studies have found that school-based programs are particularly beneficial when teachers are involved in the training and delivery of the program (Frydenberg et al., 2004; Hampel et al., 2008; Garcia et al., 2010). In addition, each session was designed to last 20 min so that it would be acceptable to implement it during school hours in the Japanese context.

We hypothesized that after implementing the MAP, the intervention group would show decreased anxiety and depression, as well as improved emotional regulation, compared with the control group.

## Materials and methods

### Research design

The study design was quasi-experimental, with three times point evaluations, where the intervention group underwent the MAP and the control group did not. The MAP is a universal program for all students, regardless of grade level. Only two schools applied to participate in this study. All assessments were performed pre-program (week 0), post-program (week 8), and at a 3-month follow-up.

### Participants

We proposed two programs to the two schools that applied for this study: One was to implement both the prevention program and the questionnaire, which would require approximately 220 min (20 min × 11 times) of class time, and the other was to administer the questionnaire only, which would require approximately 60 min (20 min × 3 times). One school was located in Tokyo and the other was located in the Kanagawa Prefecture; each school was given the liberty to choose the program according to its schedule. The study was blinded, and the schools were unaware of whether the program they selected was the intervention or the control condition.

All participants (*N* = 349) were adolescents aged 12–13 years, from nine classes in two schools at a metropolitan area in Japan. The intervention group included 176 adolescents (95 boys and 81 girls) from five classes in a private school. The control group included 172 adolescents (89 boys and 83 girls) from four classes in a national school. All students participated in the program as if it were a classroom activity. Written informed consent was obtained from the guardians of the participants, and data from students whose guardians did not provide consent were excluded from the analysis.

### Procedure

This study received ethical approval from the Ethics Committee of the Graduate School of Medicine, Chiba University (approval no: 2331). All methods were carried out in accordance with the relevant guidelines. After ethical approval, we approached school principals with an explanation of the study’s purpose and methods. Homeroom teachers were provided with a description of the purpose of the MAP, and they assisted students in completing the tasks. The first author, a counselor, served as program leader and performed the following three tasks: (1) explaining the program content to the class teachers before program implementation, (2) conducting psychoeducation and skill practice sessions for the students according to the worksheets and explaining homework assignments to the students, and (3) reviewing the homework assignments and signing and returning the submitted homework to the students.

The program was explained to the participants and a letter was sent to their parents to inform them that their child had been invited to participate in a group program to help build their emotional expression, emotional regulation, and coping skills. The program comprised eight sessions administered over 8 weeks, and started in September 2016. For the control group, a school counselor conducted the regular activities of mental health promotion, such as counseling students, at their request or upon consultation with teachers. Both groups’ data were collected simultaneously.

## Intervention

### Mindfulness awareness program

The MAP was developed by the authors, and its six features are explained below.

### Session duration

The duration of each class in the Japanese junior high schools was 50 min, and each session was designed to be completed within 20 min, that is, less than half the length of a lesson.

### Program composition

We structured the program to consist of three parts (Session 1, “Recognition and understanding of emotions”; Session 2, “Expressing and labeling emotions”; and Sessions 3–8, “Managing and regulating emotions”), with reference to previous studies aimed at improving the emotional regulation skills of Japanese students (Uchida and Yamasaki, 2012). As the main purpose of this program was to improve emotional regulation, sessions focused on how to manage and regulate emotions. The MAP is centered on mindfulness meditation for improving emotional self-control and alleviating depression and anxiety.

### Worksheet

Psychoeducation and skills practice for BCBT were provided to participants in a worksheet. Homework (skills to be practiced for the next session) was presented on the back of the worksheet.

### Program implementation

The program was conducted by a school counselor in collaboration with the homeroom teacher, who completed the worksheet in advance; the counselor asked the teacher to introduce the worksheet’s contents to promote students’ understanding. Homework submissions were collected in the next session, reviewed by the counselor, and returned to the students after all sessions were completed. Delayed homework submissions were collected by the homeroom teacher in the last session. After each session, letters were sent to all the parents to inform them of the MAP sessions’ contents. Feedback regarding the MAP was collected from the participants of the intervention group at the end of the last session. We took the following steps to maintain program fidelity in all sessions. (1) We set up a pre-session meeting to share the program’s content and procedures with homeroom teachers. (2) We discussed implementation details with the last author after the session.

### Program sessions’ contents

The aims and activities for the students in each session of the MAP were as follows: the first session’s aim was to “Recognize [and] understand emotion,” and activity was to “Select a word and write about that feeling, including its intensity and duration”; the second session’s aim was “Psychoeducation on the mind–body relationship,” and activity was to “Select an emotional event and write the body’s response to feelings”; the third session’s aim was to “Learn mindfulness meditation for emotional regulation (part one),” and activity was to “Practice mindful breathing by following instructions”; the fourth session’s aim was to “Learn mindfulness meditation for emotional regulation (part two),” and activity was to “Practice mindful breathing while visualizing a quiet place”; the fifth session’s aim was to “Learn mindfulness meditation for emotional regulation (part three),” and activity was to “Practice mindful breathing with body awareness”; the sixth session’s aim was “Psychoeducation on self-compassion,” and activity was to “Learn self-compassion. Find [their] happiness and appreciation”; the seventh session’s aim was “Understanding self-compassion,” and activity was to “Practice self-compassion. Acknowledge [their] good points and someone else’s kindness”; and the eight session’s aim was a “Review of earlier sessions and evaluation of the program,” and activity was to “Summarize what [they] learned and understood in the program.”

### Homework tasks’ contents

The homework content for each of the eight sessions and number of exercises per session were as follows: (1) “Awareness of various feelings in daily life and the situations that evoked those feelings” (6 exercises); (2) “Awareness of feelings and their intensity, as well as [their] behavior, thoughts, and physical sensations at the time” (3 exercises); (3) “Monitor the situation and [their] impressions working with mindfulness meditation” (7 exercises); (4) “Monitor the situation and [their] impressions of mindfulness meditation while visualizing a quiet place” (6 exercises); (5) “Monitor the situation and what [they] noticed as [they] engaged in mindfulness meditation with body awareness” (3 exercises); (6) “Look back on [their] day and record [their] happiness and appreciation” (4 exercises); (7) “Look back on [their] day and record [their] efforts and the kindnesses [they] received from others” (3 exercises); and (8) “Write [their] impressions of this program and answer the quiz on the key points of each session” (1 exercise).

## Measures

### Japanese version of the emotional skills and competence questionnaire

The original version of the Emotional Skills and Competence Questionnaire (ESCQ) was developed by Takšić and Inteligencije (1998), who recruited Croatian participants and based the instrument on the emotional intelligence model (Mayer and Salovery, 1997). This includes 45 items divided into the following three subscales: (1) *Perceive and Understand Emotion* (PU; e.g., “I notice when somebody feels down”), (2) *Express and Label Emotion* (EL; e.g., “I am able to express my emotions well”), and (3) *Manage and Regulate Emotion* (MR; e.g., “I try to retain a good mood”). The original version of the ESCQ indicated that the alpha coefficients of the subscales were sufficient to confirm the reliability of the questionnaire (Takšić, 2002).

Toyota et al. (2007) developed the Japanese version of the ESCQ (J-ESCQ), consisting of 21 items for junior high school students; it comprised three subscales (EL, 6 items; PU, 8 items; and MR, 7 items). The J-ESCQ has been confirmed to be reliable and valid. Further, the J-ESCQ is adjusted for middle school students by its use of plain expressions about emotional skills and competence.

This study used the J-ESCQ to assess participants’ emotional regulation at pre- (T1) and post-intervention (T2), as well as at a 3-month follow-up (T3). Participants rated each item on a four-point scale (“never,” “seldom,” “usually,” and “always”) to indicate how often they felt or thought about the statement expressed. The total J-ESCQ score ranged from 21 to 84, with higher scores indicating more emotional skills and competence. The total score and the scores of the three subscales were used to analyze emotional regulation. Cronbach’s alpha for the J-ESCQ was 0.92 (T1), 0.94 (T2), and 0.94 (T3) in this study. Cronbach’s alphas for the 3 subscales (PU, EL, and MR) were 0.89, 0.92, and 0.77 at T1, 0.92, 0.95, and 0.84 at T2, and 0.91, 0.95, and 0.85 at T3. The item-total correlations were also checked, and all items were confirmed to be > 0.3. Test–retest reliability was calculated using intraclass correlation coefficient (ICC) estimates and their 95% confidence intervals (CI)—a single measurement, absolute agreement, two-way mixed-effects model. Based on the 95% CI of the ICC estimate, values less than 0.5 indicated poor reliability, between 0.5 and 0.75 indicated moderate reliability, between 0.75 and 0.9 indicated good reliability, and greater than 0.90 indicated excellent reliability (Koo and Li, 2016). The ICC (*n* = 327) was 0.77 (95% CI: 0.73–0.80), which can be regarded as a “moderate” to “good” level of reliability.

### Depression self-rating scale for children

The Depression Self-Rating Scale for Children (DSRS-C; Birleson, 1981) is an 18-item measure of depressive symptoms in children and adolescents aged between 6 and 15 years. Murata et al. (1996) translated the English scale into Japanese. The latter version has been confirmed to be reliable and valid.

Respondents were asked to rate each item on a 3-point scale from 0 (“never”) to 2 (“always”). Birleson et al. (1987) determined the cut-off of DSRS-C as 15, but Murata et al. (1996) reported that 16 was appropriate by applying the Japanese version of the DSRS-C to Japanese children and adolescents. The DSRS-C total score ranges from 0 to 36, with higher scores indicating greater severity of depressive symptoms. The DSRS-C has two subscales, namely, *Depressive Mood* (DM; 9 items, total score 0–18) and *Decline of Activity and Enjoyment* (DAE; 9 items, total score 0–18). The DSRS-C was developed based on Zung (1965). Self-rating Depression Scale, and is characterized by its use of plainer expressions, compared with other depression scales (Sato et al., 2009). Sato et al. (2009) reported that the DSRS-C is the most recommended screening test for depression in Japanese children.

Cronbach’s alpha for the DSRS-C total score was 0.88 (T1), 0.88 (T2), and 0.89 (T3) in this study. Cronbach’s alphas for the two subscales (DM and DAE) were 0.83 and 0.83 at T1, 0.82 and 0.85 at T2, and 0.85 and 0.84 at T3. These values are large enough to ensure the reliability of DSRS-C. The item-total correlations were also checked, and all items were confirmed to be > 0.3. The ICC (*n* = 326) was 0.77 (95% CI: 0.73–0.80), which can be regarded as a “moderate” to “good” level of reliability.

### Spence children’s anxiety scale

The Spence Children’s Anxiety Scale (SCAS) is used for assessing symptoms of anxiety disorders among children and adolescents (Spence, 1998). The scale provides an overall measure of total anxiety, as well as six subtype scores, which correspond to the DSM-IV-TR anxiety disorder categories: separation anxiety (SAD), social phobia (SoP), obsessive compulsive (OCD), panic attack and agoraphobia (PAA), general anxiety (GAD), and fear of physical injury (Pij). The SCAS has been regarded as a useful measure to assess child anxiety symptoms in different countries, including non-Western and non-English speaking countries (Ishikawa et al., 2009). The SCAS is a reliable instrument for cross-cultural use; the original six-factor model is appropriate for cross-cultural application (Orgilés et al., 2016).

The SCAS is a 38-item measure of anxiety symptoms in children and adolescents aged 8–15 years. Respondents were asked to rate each item on a 4-point scale in terms of its frequency, ranging from 0 (“never”) to 3 (“always”). The total score ranges from 0 to 114, with higher scores indicating greater severity of anxiety symptoms. Ishikawa et al. (2009) confirmed the validity and reliability of the translated Japanese version of the SCAS.

Cronbach’s alpha for the SCAS total score was 0.93 (T1), 0.94 (T2), and 0.93 (T3) in this study. Cronbach’s alphas for the six subscales (SAD, SoP, OCD, PAA, Pij, and GAD) were 0.71, 0.76, 0.63, 0.86, 0.54 and 0.81 at T1; 0.77, 0.79, 0.73, 0.88, 0.59 and 0.80 at T2; and 0.76, 0.79, 0.71, 0.85, 0.57, and 0.81 at T3. In this study, the internal consistency of Pij was relatively low (compared with other subscales) but only somewhat lower or comparable to that in the original study (*α* = 0.6; Spence, 1998). The consistency of Pij was lower than that of other subscales because Pij includes various objects that can arouse children’s fear; however, items of other anxiety symptoms also refer to similar content (Ishikawa et al., 2009). The internal consistency for Pij was less than 0.70 and should be interpreted with caution. The item-total correlations were also evaluated, and all items were confirmed to be > 0.3 except for Q37 (*r* = 0.213): “I have to do some things in just the right way to stop bad things happening.” The ICC (*n* = 325) was 0.82 (95% CI: 0.79–0.85), which can be regarded as a “good” level of reliability.

### Data collection

At each of the time points (before the start of the MAP, after the end of the MAP, and 3 months after the end of the MAP), the students were asked to answer each measurement form in an envelope distributed by their homeroom teacher in the classroom. When the questionnaires were collected, they were returned to the envelopes so that other students would not know what they had answered.

### Feedback questionnaire

The intervention group participants completed a survey in the last session of the program. Students rated the following three questions on a 1–7-point Likert scale (1 = “very little,” 4 = “neither,” and 7 = “completely”), with higher scores indicating greater satisfaction and activity levels: (1) “How would you rate the program in terms of being enjoyable?” (termed “Enjoy”; 2) “How easy to understand do you think the program is? (termed “E to U”; 3) “How much effort did you put into your homework?” (named “HW Effort”).

### Homework score

The intervention group participants worked on homework as part of the program. The scores from exercises worked on as homework (called “HW score”) were used for statistical analysis. Homework scores were given based on the number of exercises a student completed in their homework. The HW scores ranged from 0 to 33, where higher scores indicated increased compliance with homework.

### Data analysis

The Pearson chi-square test was used to compare baseline categorical data between the groups. To examine group differences at T1, a *t*-test was conducted for the three scales, and Cohen’s d statistic (Cohen, 1988) was used to calculate effect sizes. We assessed item-total correlations (< 0.3) and Cronbach’s alpha to determine the internal consistency of each questionnaire. Test–retest reliability was calculated using ICC estimates and their 95% CIs—a single measurement, absolute agreement, two-way mixed-effects model.

A mixed model was used to investigate differences in groups and gender differences in outcome variables at the three time points. The appropriateness of the mixed model was first evaluated through an examination of the extent to which variance is explained within and between classes by the ICC calculation.

The mixed model considers the clustering of repeated measurements of individuals. The interindividual variability of the intercept was considered using a random intercept model. The baseline score differences between the intervention and control groups were included in the model as fixed effects. In terms of intraclass similarity, the number of classes was small, and the ICC value was low, between 0.009 and 0.04. Thus, this analysis did not consider the clustering of classes. These interaction terms were included in the model to examine differences in the outcome variables and trajectories according to group and gender. The dependent variables were the results obtained for each scale.

Missing data were handled *via* full information maximum likelihood estimation. The explanatory variables were gender (boys vs. girls), time points (T1, T2, and T3), and group (intervention vs. control).

The Wilcoxon rank-sum test was used to investigate gender differences in HW scores and outcomes of the feedback questionnaire. The effect size (Cohen’s *r*) was calculated. Following Cohen (1988) suggestion, an *r* of 0.10 was interpreted as small, 0.30 as medium, and > 0.50 as large.

Analyses were conducted with significance set at *p* < 0.05. All data analyses were performed using Stata software version 14.2 (Stata Corp LLC, College Station, TX, United States). All authors were involved in data verification and analysis.

## Results

### Participant data

**Figure 1:**
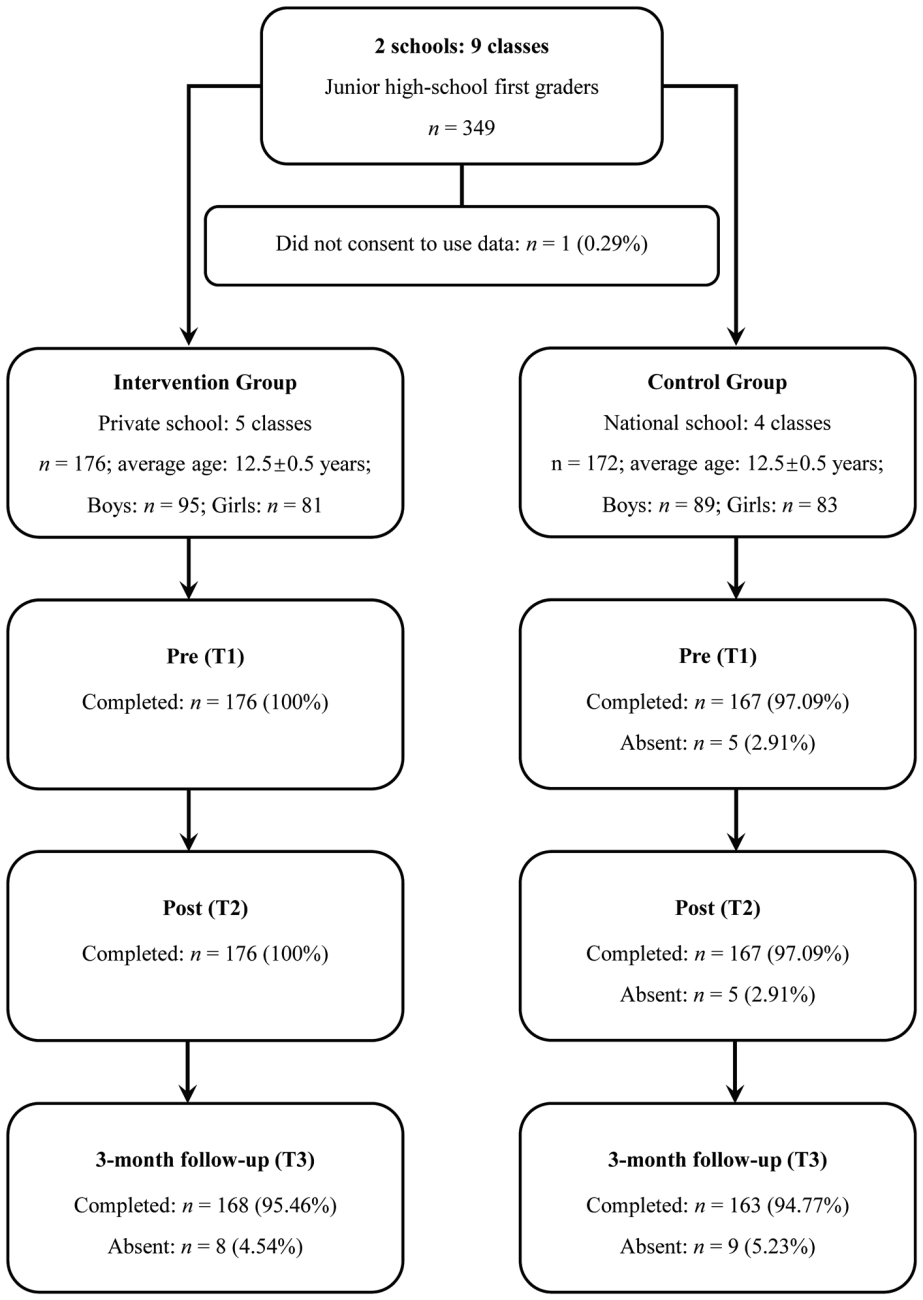
Flowchart of participant recruitment and retention.

Figure 1 shows a flowchart of the recruitment and retention of study participants. One student (0.29%) was excluded from the analysis, as parental consent for use of their data was not obtained. From nine classes in the two schools, 348 students participated. In the intervention group, no participant was absent at T1 and T2, compared with five participants (2.99%) in the control group at both T1 and T2. At T3, eight participants (4.54%) in the intervention group and nine (5.23%) in the control group were absent.

### Pre-intervention comparison

Table 1 shows the outcome measurements and number of valid responses at each time point by gender. An analysis of differences in the baseline gender ratio revealed no significant differences between the intervention and control groups [*χ*^2^(1) = 0.22, *p* = 0.64]. An analysis of differences in age by gender at baseline showed no significant differences between the groups for neither boys [*t*(181) = 0.83, *p* = 0.41] nor girls [*t*(162) = −0.96, *p* = 0.34]. The average ages of the intervention and control groups were 12.53 [standard deviation (*SD*) = 0.50, range = 12–13] and 12.53 (*SD* = 0.50, range = 12–13) years, respectively.

**Table 1:** Outcome measurements and the number of valid responses at the three time points by gender.

Girls			T1(Pre)				T2(Post)				T3(Follow-up)			
			*n* (%)		M (SD)		*n* (%)		M (SD)		*n* (%)		M (SD)	
J-ESCQ	Intervention	(*n* = 81)	81	(100.)	59.89	(10.55)	81	(100.0)	58.63	(10.19)	77	(95.06)	60.43	(10.68)
	Control	(*n* = 83)	79	(95.18)	63.33	(10.45)	82	(98.80)	63.32	(11.28)	78	(93.98)	62.67	(11.05)
	Total	(*n* = 164)	160	(97.56)	61.59	(10.41)	163	(99.39)	60.99	(10.97)	155	(94.51)	61.55	(10.89)
EL					16.36	(4.76)			15.80	(4.39)			16.69	(4.33)
					17.27	(4.51)			17.38	(4.83)			17.24	(4.85)
					16.81	(4.64)			16.60	(4.67)			16.97	(4.59)
PU					23.02	(4.62)			22.59	(4.67)			22.77	(4.53)
					24.22	(4.50)			24.57	(4.52)			24.78	(4.22)
					23.61	(4.59)			23.59	(4.69)			23.78	(4.48)
MR					20.51	(3.98)			20.23	(4.15)			20.97	(4.29)
					21.85	(3.85)			21.37	(3.93)			20.64	(4.14)
					21.17	(3.96)			20.80	(4.07)			20.81	(4.20)
DSRS-C	Intervention	(*n* = 81)	81	(100.)	11.44	(7.47)	81	(100.0)	11.26	(5.78)	77	(95.06)	10.12	(6.29)
	Control	(*n* = 83)	79	(95.18)	10.22	(6.25)	82	(98.80)	9.83	(6.71)	78	(93.98)	10.41	(6.63)
	Total	(*n* = 164)	160	(97.56)	10.84	(6.90)	163	(99.39)	10.54	(6.29)	155	(94.51)	10.26	(6.45)
DM					4.26	(3.64)			4.14	(3.09)			3.62	(3.21)
					3.95	(3.02)			4.20	(3.48)			4.14	(3.56)
					4.11	(3.34)			4.17	(3.28)			3.88	(3.39)
DAE					7.19	(4.50)			7.12	(3.68)			6.49	(3.92)
					6.27	(3.85)			5.63	(3.98)			6.54	(3.49)
					6.74	(4.20)			6.37	(3.69)			6.52	(3.70)
SCAS	Intervention	(*n* = 81)	81	(100.)	30.21	(21.50)	81	(100.0)	28.33	(21.33)	77	(95.06)	26.19	(19.63)
	Control	(*n* = 83)	79	(95.18)	30.58	(17.49)	82	(98.80)	27.37	(18.91)	78	(93.98)	26.88	(17.07)
	Total	(*n* = 164)	160	(97.56)	30.39	(19.56)	163	(99.39)	27.85	(20.10)	155	(94.51)	26.54	(18.33)
SAD					3.49	(3.70)			3.19	(3.62)			3.13	(3.51)
					4.03	(3.19)			3.70	(3.50)			3.10	(2.76)
					3.76	(3.46)			3.44	(3.56)			3.12	(3.14)
SoP					6.49	(4.52)			6.23	(4.49)			6.04	(4.37)
					6.38	(4.33)			6.07	(4.43)			5.82	(4.16)
					6.44	(4.41)			6.15	(4.44)			5.92	(4.25)
OCD					5.05	(3.92)			4.69	(3.75)			4.09	(3.45)
					5.35	(3.09)			4.56	(3.39)			4.60	(3.52)
					5.20	(3.53)			4.63	(3.56)			4.35	(3.48)
PAA					4.35	(5.28)			4.07	(4.97)			3.43	(4.36)
					4.50	(4.78)			3.85	(4.85)			3.83	(4.55)
					4.43	(5.04)			3.96	(4.90)			3.63	(4.45)
Pij					4.74	(3.12)			4.41	(3.36)			4.29	(3.12)
					4.81	(3.11)			4.06	(2.96)			4.55	(3.03)
					4.78	(3.11)			4.23	(3.16)			4.42	(3.07)
GAD					6.09	(4.74)			5.74	(4.22)			5.22	(4.35)
					5.51	(3.63)			5.12	(4.07)			4.97	(4.16)
					5.80	(4.40)			5.43	(4.14)			5.10	(4.25)
**Boys**			**T1(Pre)**				**T2(Post)**				**T3(Follow-up)**			
			***n* (%)**		**M (SD)**		***n* (%)**		**M (SD)**		***n* (%)**		**M (SD)**	
J-ESCQ	Intervention	(*n* = 95)	95	(100)	57.89	(10.16)	95	(100)	55.51	(11.82)	91	(95.79)	56.89	(11.27)
	Control	(*n* = 89)	88	(98.88)	62.07	(11.94)	85	(95.51)	64.24	(11.98)	85	(95.51)	63.60	(12.19)
	Total	(*n* = 184)	183	(99.46)	59.70	(11.44)	180	(97.83)	59.63	(12.65)	176	(95.65)	60.13	(12.16)
EL					16.43	(4.20)			15.57	(4.23)			15.79	(4.07)
					17.39	(4.35)			18.05	(4.48)			17.95	(4.36)
					16.89	(4.29)			16.74	(4.51)			16.84	(4.34)
PU					21.25	(4.82)			20.99	(5.37)			21.44	(4.98)
					23.17	(5.40)			24.31	(5.48)			23.98	(5.11)
					22.17	(5.18)			22.56	(5.65)			22.66	(5.19)
MR					19.82	(3.95)			18.95	(4.41)			19.66	(4.18)
					21.51	(4.28)			21.88	(4.48)			21.67	(4.94)
					20.63	(4.19)			20.33	(4.67)			20.63	(4.66)
DSRS-C	Intervention	(*n* = 95)	95	(100)	12.08	(6.43)	95	(100)	12.89	(6.90)	90	(94.74)	12.16	(7.11)
	Control	(*n* = 89)	88	(98.88)	9.81	(6.03)	85	(95.51)	9.20	(5.94)	85	(95.51)	9.01	(5.91)
	Total	(*n* = 184)	183	(99.46)	10.99	(6.33)	180	(97.83)	11.15	(6.71)	175	(95.11)	10.63	(6.72)
DM					3.88	(3.17)			3.93	(3.10)			3.80	(3.42)
					3.64	(3.04)			3.42	(2.81)			3.00	(2.83)
					3.77	(3.10)			3.69	(2.97)			3.41	(3.16)
DAE					8.20	(4.31)			8.97	(4.73)			8.36	(4.71)
					6.17	(3.80)			5.78	(3.82)			5.96	(3.51)
					7.23	(4.15)			7.49	(4.47)			7.19	(4.33)
SCAS	Intervention	(*n* = 95)	95	(100)	24.60	(17.62)	95	(100)	25.07	(18.57)	90	(94.74)	24.04	(18.75)
	Control	(*n* = 89)	88	(98.88)	23.75	(14.90)	85	(95.51)	21.27	(17.00)	85	(95.51)	20.06	(15.42)
	Total	(*n* = 184)	183	(99.46)	24.19	(16.32)	180	(97.83)	23.28	(17.90)	175	(95.11)	22.11	(17.28)
SAD					3.06	(3.30)			2.94	(3.33)			2.92	(3.52)
					3.17	(2.80)			2.49	(2.75)			2.26	(2.70)
					3.12	(3.06)			2.73	(3.07)			2.60	(3.16)
SoP					5.51	(3.98)			5.54	(4.11)			4.94	(3.99)
					3.17	(2.80)			2.49	(2.75)			2.26	(2.70)
					5.08	(3.82)			4.61	(3.88)			4.42	(3.88)
OCD					4.88	(3.22)			5.02	(3.68)			5.02	(3.55)
					3.17	(2.80)			2.49	(2.75)			2.26	(2.70)
					5.00	(3.13)			4.86	(3.77)			4.73	(3.47)
PAA					3.22	(4.64)			3.62	(4.59)			3.37	(4.56)
					2.90	(4.04)			2.82	(4.25)			2.29	(3.27)
					3.07	(4.35)			3.24	(4.44)			2.85	(4.01)
Pij					3.31	(3.11)			2.93	(3.02)			2.80	(2.95)
					3.50	(2.36)			3.54	(2.78)			3.51	(2.97)
					3.40	(2.76)			3.22	(2.91)			3.14	(2.97)
GAD					4.61	(3.63)			5.03	(3.76)			4.99	(3.81)
					4.44	(3.49)			4.16	(3.47)			3.72	(3.41)
					4.53	(3.55)			4.62	(3.64)			4.37	(3.66)

M, mean; SD, standard Deviation; J-ESCQ, Japanese version of the Emotional Skills and Competence Questionnaire; PU, Perceive and Understand Emotion; EL, Express and Label Emotion; MR, Manage and Regulate Emotion; DSRS-C, Depression Self-Rating Scale for Children; DM, Depressive Mood; DAE, Decline of Activity and Enjoyment; SCAS, Spence Children’s Anxiety Scale; SAD, Separation Anxiety Disorder; SoP, Social Phobia; OCD, Obsessive–Compulsive Disorder; PAA, Panic Attack and Agoraphobia; Pij, Physical Injury Fear; GAD, Generalized Anxiety Disorder.

Regarding the characteristics of outcome measures between the intervention and control groups at baseline, there was a significant difference in the overall J-ESCQ scores [*t*(181) = 2.74, *p* = 0.007, *d* = 0.41] and in the PU [*t*(181) = 2.54, *p* = 0.01, *d* = 0.38] and MR [*t*(181) = 2.78, *p* = 0.006, *d* = 0.41] subscale scores in the J-ESCQ at baseline among boys. Among girls, there was a significant difference between the intervention and control groups in the overall J-ESCQ scores [*t*(158) = 2.11, *p* = 0.04, *d* = 0.33] and MR [*t*(158) = 2.17, *p* = 0.03, *d* = 0.34] at baseline, as well as in the baseline overall DSRS-C scores [*t*(181) = −2.47, *p* = 0.02, *d* = −0.37] and in the DAE [*t*(181) = −3.37, *p* = 0.001, *d* = −0.50] subscale among boys. The baseline score differences between the intervention and control groups were included in the model as fixed effects.

### Intervention outcomes

**Figure 2:**
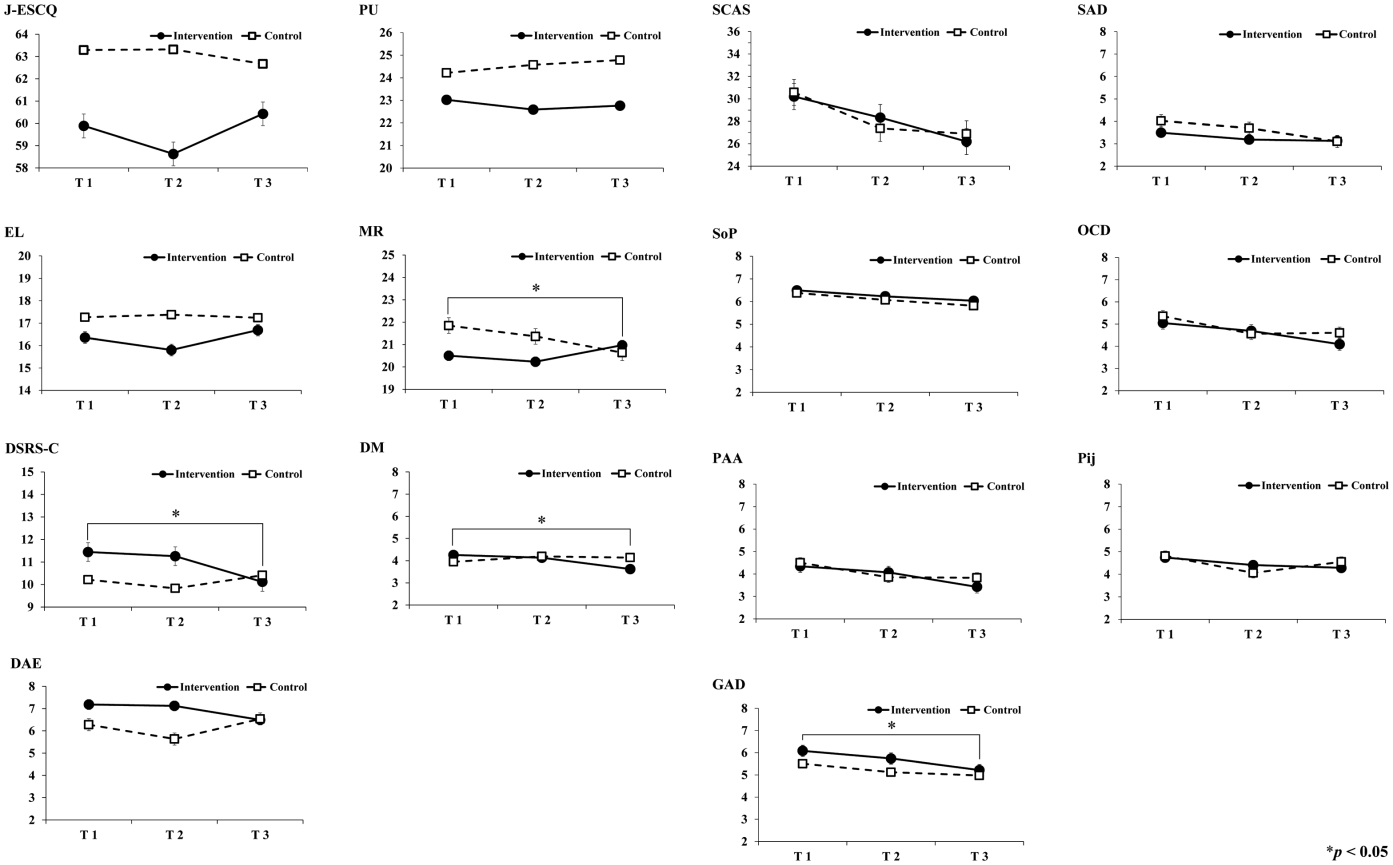
Comparisons of female participants’ mean scores at the three time points. T1, Pre-intervention; T2, Post-intervention; T3, Follow-up; J-ESCQ, Japanese version of the Emotional Skills and Competence Questionnaire; PU, Perceive and Understand Emotion; EL, Express and Label Emotion; MR, Manage and Regulate Emotion; DSRS-C, Depression Self-Rating Scale for Children; DM, Depressive Mood; DAE, Decline of Activity and Enjoyment; SCAS, The Spence Children’s Anxiety Scale; SAD, Separation Anxiety Disorder; SoP, Social Phobia; OCD, Obsessive–Compulsive Disorder; PAA, Panic Attack and Agoraphobia; Pij, Physical Injury Fear; GAD, Generalized Anxiety Disorder. *Error bars* indicate Standard Error.

Table 2 shows the results for each variable in the mixed-effects model for *group*, *time*, and *gender* (i.e., the main effects of *group*, *time*, and *gender,* as well as the between-factor interaction for *group* and *time*, as well as for *group*, *time*, and *gender*). In the analysis, the reference category used for *group* was *control* (control, intervention), for *time* was T1 (T1, T2, T3), and for *gender* was *boys* (boys, girls). Figure 2 depicts the comparisons of female participants’ mean scores at the three time points.

**Table 2:** Outcomes of each variable in the mixed-effects model for group, time, and gender.

			Coef	SE	*p*	95% Confidence interval	
J-ESCQ	Group	*Intervention*	−0.586	0.920	0.524	−2.389	1.217
	Time	*T2*	1.691	0.835	**0.043**	0.053	3.328
		*T3*	1.008	0.838	0.229	−0.636	2.651
	Gender	*Girls*	0.162	0.959	0.866	−1.717	2.041
	Group*Time	*T2*	−3.691	1.154	**0.001**	−5.952	−1.429
		*T3*	−1.859	1.163	0.110	−4.138	0.420
	Group*Time*Gender	*T2*	2.286	1.685	0.175	−1.018	5.589
		*T3*	3.276	1.702	0.054	−0.060	6.612
EL	Group	*Intervention*	−0.246	0.439	0.575	−1.107	0.615
	Time	*T2*	0.512	0.396	0.196	−0.264	1.289
		*T3*	0.420	0.398	0.291	−0.359	1.200
	Gender	*Girls*	−0.031	0.459	0.946	−0.931	0.869
	Group*Time	*T2*	−1.375	0.547	**0.012**	−2.448	−0.303
		*T3*	−1.156	0.552	**0.036**	−2.237	−0.074
	Group*Time*Gender	*T2*	0.537	0.799	0.502	−1.030	2.104
		*T3*	1.445	0.807	0.074	−0.138	3.027
PU	Group	*Intervention*	−0.331	0.416	0.427	−1.146	0.485
	Time	*T2*	1.000	0.387	**0.010**	0.241	1.758
		*T3*	0.668	0.388	0.085	−0.092	1.429
	Gender	*Girls*	0.180	0.434	0.678	−0.671	1.031
	Group*Time	*T2*	−1.263	0.534	**0.018**	−2.310	−0.216
		*T3*	−0.554	0.538	0.304	−1.609	0.501
	Group*Time*Gender	*T2*	0.515	0.780	0.509	−1.014	2.045
		*T3*	0.046	0.788	0.954	−1.499	1.590
MR	Group	*Intervention*	−0.254	0.377	0.500	−0.993	0.485
	Time	*T2*	0.207	0.348	0.553	−0.476	0.889
		*T3*	−0.054	0.349	0.878	−0.739	0.631
	Gender	*Girls*	0.051	0.393	0.897	−0.719	0.820
	Group*Time	*T2*	−1.080	0.481	**0.025**	−2.023	−0.138
		*T3*	−0.166	0.485	0.733	−1.116	0.784
	Group*Time*Gender	*T2*	1.251	0.703	0.075	−0.126	2.639
		*T3*	1.782	0.709	**0.012**	0.392	3.172
DSRS-C	Group	*Intervention*	0.555	0.617	0.369	−0.654	1.763
	Time	*T2*	−0.373	0.467	0.425	−1.288	0.543
		*T3*	−0.468	0.469	0.319	−1.387	0.452
	Gender	*Girls*	0.286	0.643	0.656	−0.671	1.546
	Group*Time	*T2*	1.183	0.645	0.066	−0.080	2.446
		*T3*	0.605	0.651	0.353	−0.671	1.882
	Group*Time*Gender	*T2*	−0.444	0.941	0.637	−2.289	1.401
		*T3*	−2.174	0.952	**0.022**	−4.041	−0.308
DM	Group	*Intervention*	−0.355	0.314	0.258	−0.970	0.260
	Time	*T2*	−0.109	0.248	0.661	−0.594	0.377
		*T3*	−0.477	0.249	0.055	−0.964	0.010
	Gender	*Girls*	0.158	0.328	0.629	−0.484	0.801
	Group*Time	*T2*	0.151	0.342	0.659	−0.519	0.820
		*T3*	0.494	0.345	0.152	−0.182	1.171
	Group*Time*Gender	*T2*	−0.245	0.499	0.624	−1.223	0.733
		*T3*	−1.291	0.505	**0.011**	−2.281	−0.302
DAE	Group	*Intervention*	1.859	0.505	**0.000**	0.869	2.849
	Time	*T2*	−0.228	0.332	0.493	−0.879	0.424
		*T3*	−0.067	0.334	0.842	−0.721	0.588
	Gender	*Girls*	−0.104	0.529	0.844	−1.142	0.933
	Group*Time	*T2*	0.996	0.458	**0.030**	0.098	1.895
		*T3*	0.233	0.464	0.615	−0.675	1.142
	Group*Time*Gender	*T2*	−0.189	0.669	0.777	−1.502	1.122
		*T3*	−1.244	0.678	0.066	−2.572	0.840
SCAS	Group	*Intervention*	0.091	1.262	0.946	−2.382	2.563
	Time	*T2*	−1.878	1.139	0.099	−4.111	0.355
		*T3*	−3.111	1.143	**0.007**	−5.352	−0.870
	Gender	*Girls*	0.732	1.324	0.580	−1.863	3.327
	Group*Time	*T2*	1.974	1.577	0.211	−1.117	5.065
		*T3*	2.844	1.593	0.074	−0.277	5.965
	Group*Time*Gender	*T2*	0.845	2.301	0.713	−3.664	5.355
		*T3*	−2.919	2.325	0.200	−7.537	1.579
SAD	Group	*Intervention*	−0.020	0.268	0.942	−0.545	0.506
	Time	*T2*	−0.622	0.243	**0.010**	−1.098	−0.147
		*T3*	−0.860	0.243	**0.000**	−1.337	−0.383
	Gender	*Girls*	0.157	0.281	0.576	−0.393	0.708
	Group*Time	*T2*	0.463	0.336	0.168	−0.195	1.121
		*T3*	0.643	0.339	0.058	−0.022	1.307
	Group*Time*Gender	*T2*	−0.220	0.490	0.653	−1.180	0.740
		*T3*	−0.030	0.495	0.952	−1.000	0.940
SoP	Group	*Intervention*	0.163	0.339	0.630	−0.501	0.828
	Time	*T2*	−0.981	0.310	**0.002**	−1.590	−0.373
		*T3*	−0.679	0.311	**0.029**	−1.290	−0.069
	Gender	*Girls*	0.322	0.355	0.365	−0.375	1.018
	Group*Time	*T2*	0.928	0.430	**0.031**	0.086	1.770
		*T3*	0.245	0.434	0.572	−0.605	1.096
	Group*Time*Gender	*T2*	−0.536	0.627	0.393	−1.765	0.693
		*T3*	−0.226	0.633	0.721	−1.468	1.015
OCD	Group	*Intervention*	−0.037	0.300	0.901	−0.626	0.551
	Time	*T2*	−0.418	0.275	0.128	−0.867	0.120
		*T3*	−0.692	0.276	**0.012**	−1.233	−0.152
	Gender	*Girls*	0.035	0.314	0.910	−0.580	0.650
	Group*Time	*T2*	0.525	0.381	0.168	−0.221	1.271
		*T3*	0.881	0.384	**0.022**	0.128	1.634
	Group*Time*Gender	*T2*	0.187	0.555	0.736	−0.901	1.275
		*T3*	−0.983	0.561	0.080	−2.082	0.117
PAA	Group	*Intervention*	0.058	0.349	0.867	−0.625	0.742
	Time	*T2*	0.150	0.328	0.648	−0.493	0.792
		*T3*	−0.360	0.329	0.274	−1.004	0.285
	Gender	*Girls*	0.288	0.366	0.431	−0.429	1.005
	Group*Time	*T2*	0.137	0.454	0.762	−0.752	1.027
		*T3*	0.599	0.458	0.191	−0.299	1.497
	Group*Time*Gender	*T2*	0.557	0.662	0.400	−0.741	1.856
		*T3*	−0.731	0.669	0.275	−2.042	0.581
Pij	Group	*Intervention*	−0.027	0.240	0.911	−0.497	0.443
	Time	*T2*	0.069	0.216	0.750	−0.354	0.491
		*T3*	−0.052	0.216	0.809	−0.476	0.372
	Gender	*Girls*	0.184	0.252	0.465	−0.310	0.678
	Group*Time	*T2*	−0.494	0.298	0.098	−1.079	0.091
		*T3*	−0.426	0.301	0.157	−1.017	0.164
	Group*Time*Gender	*T2*	0.895	0.435	**0.040**	0.041	1.748
		*T3*	0.201	0.440	0.648	−0.661	1.063
GAD	Group	*Intervention*	0.258	0.296	0.931	−0.555	0.607
	Time	*T2*	−0.109	0.271	0.689	−0.640	0.423
		*T3*	−0.496	0.272	0.068	−1.029	0.038
	Gender	*Girls*	0.168	0.310	0.589	−0.441	0.776
	Group*Time	*T2*	0.449	0.375	0.232	−0.287	1.185
		*T3*	0.898	0.379	**0.018**	0.156	1.641
	Group*Time*Gender	*T2*	−0.061	0.548	0.911	−1.135	1.012
		*T3*	−1.183	0.553	**0.033**	−2.267	−0.098

For the reference category, Control in Group (Control, Intervention), T1 in Time (T1, T2, T3), and Boys in Gender (Boys, Girls) was used. Coef.: coefficient; SE: standard error; J-ESCQ: Japanese version of the Emotional Skills and Competence Questionnaire; PU: Perceive and Understand Emotion; EL: Express and Label Emotion; MR: Manage and Regulate Emotion; DSRS-C: Depression Self-Rating Scale for Children; DM: Depressive Mood; DAE: Decline of Activity and Enjoyment; SCAS: Spence Children’s Anxiety Scale; SAD: Separation Anxiety Disorder; SoP: Social Phobia; OCD: Obsessive–Compulsive Disorder; PAA: Panic Attack and Agoraphobia; Pij: Physical Injury Fear; GAD: Generalized Anxiety Disorder, Bold: *p* < 0.05.

#### Japanese version of the ESCQ

The interaction between the factors *group* (intervention, control) and *time* (baseline: T1; post-intervention: T2; follow-up: T3) was examined. In the J-ESCQ, the interaction was significant only at T2, with an increase in the control group’s scores and a decrease in those of the intervention group, compared with T1 (*β* = −3.691, 95% CI: −5.952, −1.429, *p* = 0.001). In EL, the interaction was significant at T2 and T3, wherein the control group’s scores increased, compared with T1, while those of the intervention group decreased (T2; *β* = −1.375, 95% CI: −2.448, −0.303, *p* = 0.012 and T3; *β* = −1.156, 95% CI: −2.237, −0.074, *p* = 0.036). In PU, the interaction was significant only at T2, wherein the control group’s scores increased, while those of the intervention group decreased, compared with T1 (*β* = −1.263, 95% CI: −2.310, −0.216, *p* = 0.018). In MR, the interaction was significant only at T2, wherein the intervention group’s scores decreased more than those of the control group (*β* = −1.080, 95% CI: −2.023, −0.138, *p* = 0.025).

The interaction effects among *group*, *time,* and *gender* (boys, girls) were examined. The interaction was non-significant for the overall J-ESCQ, EL, and PU. In MR, the interaction was significant only at T3, and among boys, while the intervention group’s scores decreased more than those of the control group, compared with T1. In girls, the scores of the control group decreased, while those of the intervention group increased, compared with T1 (*β* = 1.782, 95% CI: 0.392, 3.172, *p* = 0.012).

#### Depression self-rating scale for children

The interaction between the factors *group* and *time* was examined. The interaction was not significant for the overall DSRS-C and DM. In DAE, the interaction was significant only at T2, wherein the control group exhibited lower scores, while the intervention group exhibited higher scores, compared with T1 (*β* = 0.996, 95% CI: 0.098, 1.895, *p* = 0.030).

The interaction among *group*, *time*, and *gender* was examined. The interaction was not significant for DAE. In the overall DSRS-C, the interaction was significant only at T3, and in boys; the control group’s scores decreased, while those of the intervention group increased, compared with T1. In girls, the control group’s scores increased, while those of the intervention group decreased, compared with T1 (*β* = −2.174, 95% CI: −4.041, −0.308, *p* = 0.022). In DM, the interaction was significant only at T3, and in boys; the control group’s scores decreased, while those of the intervention group increased, compared with T1. In girls, the control group’s scores increased, while those of the intervention group decreased, compared with T1 (*β* = −1.291, 95% CI: −2.181, −0.302, *p* = 0.011).

#### Spence children’s anxiety scale

The interaction between *group* and *time* was examined. The interaction was not significant for the overall SCAS, SAD, PAA, and Pij. In SoP, the interaction was significant only at T2, wherein the control group’s scores decreased more than those of the intervention group, compared with T1 (*β* = 0.928, 95% CI: 0.086, 1.770, *p* = 0.031). In OCD, the interaction was significant only at T3, wherein the control group’s scores decreased more than those of the intervention group, compared with T1 (*β* = 0.881, 95% CI: 0.128, 1.634, *p* = 0.022). In GAD, the interaction was significant only at T3, wherein the control group’s scores decreased more than those of the intervention group, compared with T1 (*β* = 0.898, 95% CI: 0.156, 1.641, *p* = 0.018).

The interaction among *group*, *time*, and *gender* was examined. The interaction was not significant for the overall SCAS, SAD, SoP, OCD, and PAA. In Pij, the interaction was significant only for boys at T2, wherein the control group’s scores increased, while those of the intervention group deceased, compared with T1. In girls, the control group’s score decreased more than that of the intervention group, compared with T1 (*β* = 0.895, 95% CI: 0.041, 1.748, *p* = 0.040). In GAD, the interaction was significant for boys only at T3, wherein the control group’s scores decreased, while those of the intervention group increased, compared with T1. In girls, the intervention group’s scores decreased more than those of the control group, compared with T1 (*β* = −1.183, 95% CI: −2.267, −0.098, *p* = 0.033).

### Feedback questionnaire on MAP and homework scores

Table 3 shows the outcomes of the MAP feedback questionnaire and HW scores in terms of gender. There were no significant differences between boys and girls for “Enjoy” and “E to U.” The scores were significantly higher for girls than for boys in “HW effort” (*z* = −4.32, *p* < 0.001, *r* = −0.33) in the MAP feedback questionnaire and homework (*z* = −3.46, *p* = 0.001, *r* = −0.26) in the HW score.

**Table 3:** Statistical outcomes on MAP feedback questionnaire and homework scores.

	Boys			Girls			Range	*z*	*p*	ES (*r*)
	*n*	M	(SD)	*n*	M	(SD)				
Enjoy	87	4.517	(1.454)	81	4.617	(1.529)	1–7	−0.480	0.631	−0.034
E to U	87	5.287	(1.363)	81	5.506	(1.370)	1–7	−1.117	0.264	−0.080
HW effort	87	3.747	(1.440)	81	4.765	(1.477)	1–7	−4.324	**0.000**	−0.330
HW Scores	94	22.968	(10.003)	82	27.683	(6.733)	0–32	−3.456	**0.001**	−0.267

MAP, Mindfulness Awareness Program; Enjoy, Outcome of “How would you rate the program in terms of being enjoyable?”; E to U, Outcome of “How much do you think the program is easy to understand?”; HW Effort, Outcome of “How much effort did you put into your homework?”; HW Scores, Number of completed exercises in homework sheets; M, mean; SD, standard deviation; ES, effect size, Bold: p < 0.05.

## Discussion

This study examined the effect of a universal mindfulness-based BCBT preventive intervention on the mental health of junior high school students. We developed a MAP structured around mindfulness for improving emotional regulation skills, while adopting the BCBT framework to adapt the mental health preventive program in the Japanese educational environment for adolescents. In the data analysis, the baseline score differences between the intervention and control groups were included in the model as fixed effects. At T3, we found that emotional regulation had improved, and anxiety and depression had decreased in female participants, compared with the control group. However, in male participants, there were no significant changes in any variable.

The results of the MAP provided some encouraging evidence of a modest positive effect. The MAP is structured around mindfulness techniques to achieve emotional regulation and encourages emotional acceptance. A higher level of habitual emotional acceptance in youth has been associated with lower levels of depressive and anxiety symptoms (Weinberg and Klonsky, 2009). Werner and Gross (2010) noted that when emotions are accepted, dysfunctional reactions, such as judging or suppressing negative emotions, may be less likely. In a prior meta-analysis, acceptance of one’s emotions had the strongest negative association with depressive and anxiety symptoms, and the strongest positive association with avoidance and rumination as maladaptive emotion regulation strategies (Schäfer et al., 2017). Therefore, we believe that participation in the MAP may have increased participants’ acceptance of emotions and reduced depression and anxiety.

Our MAP can be implemented in regular classes, but the effects will vary among male and female junior high school students. One of the implications of this study is the need to differentially adapt MBIs for men and women, with emphasis on the fact that women may demonstrate a stronger response to mindfulness intervention.

Bluth et al. (2017) investigated gender differences in response to an adolescent mindfulness intervention and made two suggestions. First, the difference in responses could be due to developmental differences; adolescent girls mature earlier than boys (Steinberg and Morris, 2001) and may, therefore, have greater interest in and understanding of a program focusing on stress reduction. Another possibility is that a mindfulness intervention may be differentially effective for male and female students (e.g., Rojiani et al., 2017). Interventions aimed at modifying affective and emotion-focused coping strategies may have different impacts on boys and girls during puberty’s onset (Kang et al., 2018). As female adolescent students are more agreeable with seeking emotional support as a coping strategy, compared with their male counterparts (Mendlowitz et al., 1999), they may show greater improvements in emotional well-being with mindfulness training, which aims to improve positive emotionality and adaptive coping strategies (Kang et al., 2018).

We found a significant difference between male and female participants’ attitudes toward homework. The MAP required students to complete worksheets during class and as homework. Boys completed fewer homework assignments than girls, which may have affected our results and requires further investigation. Cammin-Nowak et al. (2013) reported two findings on homework assignments in CBT; first, therapies that incorporate homework assignments are associated with better outcomes, compared with therapies without homework; second, patients who complete homework show greater improvement, compared with those who do not (Kazantzis et al., 2000, 2010; Mausbach et al., 2010; Klingbeil et al., 2017).

Studies on depression and anxiety among Japanese adolescents have reported that female adolescents exhibit higher scores for both depression (Kawakatsu et al., 2014) and anxiety (Ishikawa et al., 2009), compared with male adolescents. This could have been associated with girls showing greater interest in mindfulness and different rates of homework completion. Given the gender differences in interest toward mindfulness, future studies should investigate the benefits of mindfulness training for both male and female students. This will be necessary to increase students’ interest in the completion of the program.

In the present study, the program had no significant effect on boys; a homeroom teacher who participated in the MAP commented that “the perceived volume of homework may have made it difficult to maintain motivation.” Rojiani et al. (2017) mentioned that gender-specific treatment outcomes may become increasingly salient for men, as they may require mindfulness interventions better matched to the particular coping styles they tend to use. In addition to adjusting the amount of homework, further research should focus on mindfulness interventions tailored to the needs of boys in Japanese junior high schools.

Significant effects among female students were observed in the follow-up period, but not immediately post-intervention. A meta-analysis of MBIs among adolescents reported the effect to be greater at follow-up, rather than immediately after the intervention (Klingbeil et al., 2017). Mindfulness theory suggests that for MBIs to be effective, they need to be continuously practiced (Kabat-Zinn, 2009). In the present study, girls put more effort into homework, compared with boys. This may be because girls were more engaged in mindfulness practice between the post-intervention and follow-up time points. However, we did not measure whether participants continued to practice mindfulness between these time points. Researchers have noted that at-home practice can lead to positive outcomes (Biegel et al., 2009). Thus, practice at home during the 3 months until follow-up could have influenced the results.

Several intervention group participants answered in the free text section of the feedback questionnaire that they had enjoyed the program. Moreover, feedback from parents indicated that the MAP gave them a chance to talk about emotions with their children. Some homeroom teachers commented on how participation in the MAP helped them understand their students. These responses suggest that the MAP is an acceptable preventive psychoeducation program in Japanese junior high schools. A meta-analysis of the results of 25 studies that used MBIs in school-based settings revealed that most studies conducted group-based interventions in a typical classroom environment during normal school hours. Additionally, interventions delivered to students in their normal classrooms can more likely be generalized to the classroom environment, and skills learned are also more likely to be used (Felver et al., 2016).

Our study had certain limitations. First, there were significant variations in homework completion rates by gender, lack of implementation fidelity data, no data on participants’ previous psychiatric history across groups, and a lack of long-term follow-up. Further, mindfulness techniques may have an effect on emotional regulation, but verification is still lacking. The lack of data on participants’ previous psychiatric history is potentially significant, given the topic and baseline differences observed in the scales used. It is necessary to continue accumulating findings and verifying which mental health prevention program elements contribute more to improving mental health among adolescents. In addition, although previous studies have examined depression and anxiety in adolescents, their relationship to emotional regulation skills needs further validation.

Second, the participants were not randomly assigned into the two groups. This limited the validity of the results obtained in our study. Based on our findings, the program needs to be revised and further intervention studies need to be conducted. In future, we would like to conduct randomized controlled trials.

Third, there was a difference in baseline scores between the groups. Moreover, only two schools were able to participate in this study. Therefore, between-group differences were adjusted for data analysis. Additionally, the two schools are in different locations. In future, we believe it is important to conduct intervention studies with more homogeneous populations.

Fourth, depression and anxiety were measured using a self-report questionnaire. Future studies should seek to replicate our findings using interview-based assessments.

Finally, only the first author implemented the program. In the next step, it is essential to validate the effectiveness of the MAP by ensuring program implementation through school counselors. Furthermore, the data in this study include pre-pandemic data, and the program content may require reexamination to adapt it for adolescents during the pandemic.

## Conclusion

In conclusion, this study examined the effects of a mindfulness-based BCBT intervention for depression, anxiety, and emotional regulation in a junior high school setting in Japan. Our results indicate the efficacy of a mindfulness-based BCBT approach in reducing depression and anxiety and enhancing emotional regulation in early adolescents. Further, the MAP appeared to be more effective in female than male adolescents.

## Data availability statement

The datasets generated during and/or analyzed during the current study are available in the OFC repository at: https://osf.io/jzgxn/?view_only=7639e92ef65748a79aa3077e8012a0c6.

## Ethics statement

The studies involving human participants were reviewed and approved by the Ethics Committee of the Graduate School of Medicine, Chiba University. Written informed consent to participate in this study was provided by the participants’ legal guardian/next of kin.

## Author contributions

KK: conceptualization, methodology, investigation, program implementation, formal analysis, and writing of original draft. YM: conceptualization, methodology, formal analysis, writing-review and editing. YH: formal analysis and writing-review and editing. All authors contributed to the article and approved the submitted version.

## Conflict of interest

The authors declare that the research was conducted in the absence of any commercial or financial relationships that could be construed as a potential conflict of interest.

## Publisher’s note

All claims expressed in this article are solely those of the authors and do not necessarily represent those of their affiliated organizations, or those of the publisher, the editors and the reviewers. Any product that may be evaluated in this article, or claim that may be made by its manufacturer, is not guaranteed or endorsed by the publisher.
